# Impact of Environmental Exposure on Infant Sleep : The Exposome Approach

**DOI:** 10.1111/jsr.70286

**Published:** 2026-02-09

**Authors:** Zeina Halbouty, Debora Tuka, Erwan Stephan‐Blanchard, Veronique Bach, Pierre Tourneux, Elodie Haraux, Karen Chardon

**Affiliations:** ^1^ PériTox Laboratory (UMR_I 01), UPJV/INERIS, Jules Verne University of Picardy Amiens France; ^2^ Neonatal Intensive Care Unit, Amiens University Hospital Amiens France; ^3^ Paediatric Surgery Unit, Amiens University Hospital Amiens France

**Keywords:** air pollution, chemical exposure, epidemiology, infant, persistent organic pollutants, sleep

## Abstract

Sleep is fundamental for infant development and health, playing a critical role in cognitive, socio‐emotional, and physical growth. However, environmental factors can impact the quality and duration of sleep in infants. This review synthesises current evidence on the associations between environmental chemical exposures and infant sleep outcomes, with a focus on the first 1000 days of life. Infants may be exposed to environmental pollutants before birth, through the placenta, or after birth, via breastfeeding, diet, and external sources such as inhalation, dust contact, or hand‐to‐mouth exposure. Given their ongoing development, foetuses and infants are particularly vulnerable to these pollutants. This period of rapid growth and maturation represents a highly sensitive window for environmental exposures. This review covers various categories of environmental pollutants, including persistent organic pollutants (PCBs, dioxins), non‐persistent pollutants (phthalates, BPA), air pollutants (particulate matter, second‐hand smoke), and water contaminants (nitrates, microplastics). Environmental chemicals exposure could be assessed using parental questionnaires or biological monitoring, while sleep is evaluated using actigraphy, polysomnography, or parental reporting. Some evidence suggests that both prenatal and postnatal exposure to environmental contaminants may be associated with sleep disturbances in children, particularly in girls. Despite the numerous studies on adults and the mechanisms associated with these pollutants (neurotoxicity, endocrine disruption), which suggest an effect on sleep, there is a lack of studies in children, resulting in limited associations in the literature. Therefore, it is imperative to conduct studies on environmental pollutants present in breast milk, diet, and/or ambient air to understand their impact on infant sleep.

## Graphical abstract

This review explores how exposure to environmental pollutants during the first 1000 days of life may affect infant sleep. Evidence suggests potential links between chemical exposures and sleep disturbances, underscoring the need for more research on early‐life vulnerability and the impact of pollutants in air, diet, and breast milk.

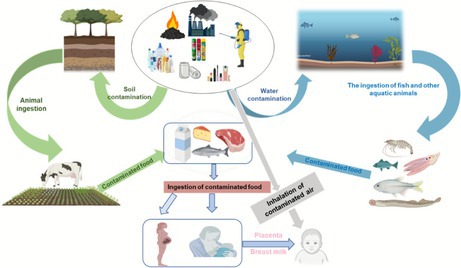

## 1 Introduction

Sleep is essential for infant development and health (Jiang 2020). The French National Institute of Health and Medical Research (INSERM) defines sleep as a state of reduced consciousness that separates two periods of wakefulness. Sleep is characterised by a loss of alertness, a decrease in muscle tone, and partial preservation of sensory perception. Numerous studies have shown the significance of sleep for cognitive, socio‐emotional, and physical growth in young children (Tham et al. 2017; Lobermeier et al. 2022). Past research has also highlighted the critical role of sleep in fundamental physiological processes such as cellular homeostasis restoration (Bathory and Tomopoulos 2017) and memory consolidation (Deak and Stickgold 2010).

Sleep is a marker of neurological development, influencing future cognitive outcomes (Gertner et al. 2002). A poor sleep quality during early childhood can alter attention and behavioural regulation (Sadeh et al. 2015), lead to emotional disturbances (Davis et al. 2004), and decrease academic performance (Chaput et al. 2016). Recent studies have linked insufficient sleep in infancy to later risk for psychopathology, including attention deficit (O'Callaghan et al. 2010), anxiety, and attention deficit hyperactivity disorder (Touchette et al. 2024). This association highlights the crucial significance of quality sleep for the mental health and overall well‐being of infants.

In the first months of life, two sleep stages could be identified: Non REM sleep stage (NREM), which alternates with rapid eye movement (REM) sleep (Peirano et al. 1993; Ardura et al. 1995). At birth, newborns do not yet have a mature circadian rhythm, and sleep is organised in ultradian cycles with a high proportion of REM sleep. Circadian organisation begins to emerge at around 3 months of age, facilitated by environmental cues such as light–dark cycles, feeding schedules, and social interactions. However, the development of sleep patterns is a gradual process that extends well beyond the first 6 months. Longitudinal studies show that across the first 3 years of life, nocturnal sleep becomes more consolidated, daytime sleep decreases progressively, and transitions between sleep stages become more organised (Louis et al. 1997; Mirmiran et al. 2003). Throughout development, changes occur, including a reduction in REM sleep and an increase in NREM sleep (Roffwarg et al. 1966; Fagioli and Salzarulo 1982). This transition is important, as it reflects the maturation from ultradian sleep cycles to more stable circadian cycles (Mirmiran et al. 2003; Korte et al. 2001).

The World Health Organisation (WHO) recommends 14–17 h of good quality sleep for infants in the first 3 months, and 12–16 h from 4 to 11 months, including naps (Organisation Mondiale de la Sante 2019). However, achieving adequate sleep duration and quality can be influenced by several external factors, including environmental exposures.

Emerging research suggests a possible link between exposure to certain environmental pollutants and sleep disturbances in infants. These pollutants include persistent organic pollutants (POPs) such as polychlorinated biphenyls, dioxins, organochlorine pesticides, brominated flame retardants, and per‐ and polyfluoroalkyl substances, as well as non‐persistent pollutants like phthalates, pyrethroids, and bisphenol A. Additionally, other environmental contaminants such as particulate matter, cigarette smoke, and water pollutants may also play a role in sleep disruption. It has been demonstrated that POPs are able to cross the placenta and subsequently be transmitted to the foetus, suggesting that early exposure to these pollutants may increase the risk of adverse developmental outcomes in children. Pollutants may affect sleep development by disrupting the central nervous system, circadian rhythms, and neurotransmitters involved in sleep regulation.

Newborns can be exposed to environmental pollutants either before birth, through the placenta (Vizcaino et al. 2014; Giaginis et al. 2012), or after birth, through breastfeeding (Lorenzetti et al. 2021), diet, and external pathways such as inhaling air, contact with dust, or hand‐to‐mouth exposure. Foetuses and infants are more sensitive to pollutants than adults. This period of maturation and growth is considered an extremely vulnerable period to environmental exposures. First, their immune system and detoxification mechanisms are not yet fully developed (Vizcaino et al. 2014). Consequently, newborns are particularly susceptible to disruptions during critical stages of development (Grandjean and Landrigan 2014; Rice and Barone 2000). Their smaller body size and lower weight, combined with their higher absorption capacity, means that minimal exposure during this period could result in adverse effects that may become apparent at a later stage (Boekelheide et al. 2012; Landrigan et al. 2003), increasing their vulnerability.

A recent systematic review (Wallace et al. 2023) on the impact of environmental factors on sleep demonstrated effects on both sleep quality and quantity in adults. This review highlighted associations between specific chemical pollutants—such as particulate matter, pesticides, dioxins, and heavy metals—and impaired sleep outcomes, including reduced sleep quality, insomnia, and sleep‐disordered breathing. Although findings remain inconsistent, the available evidence suggests that environmental exposures may be critical determinants of sleep health, underscoring the need for further research, particularly during early development. However, the impact of environmental factors on newborns remains largely unexplored. A comprehensive review is needed to evaluate the impact of environmental exposures on the sleep of newborns and infants, and highlight what needs to be explored further.

This review aims to present and synthesise the current literature investigating the associations between various chemical environmental exposures, including POPs, non‐POPs, and other environmental contaminants, on infant sleep. We initially included studies that provided data on infant sleep patterns during the first 1000 days of life. However, when we found particularly interesting information, we extended our analysis to include older children. Non‐human models (animal studies, in vitro, modelling, etc.) were not included in this review.

Sleep can be evaluated using a variety of techniques. In this review, we have included all methods to assess infant sleep patterns, including actigraphy, polysomnography (PSG), and parental questionnaires. Actigraphy is a non‐invasive method that monitors movements to determine sleep and wake cycles. PSG provides a detailed analysis of sleep architecture by measuring physiological parameters such as brain activity, eye movements, muscle activity, and breathing patterns. Questionnaires are subjective assessments of sleep quality or sleep problems, but they are easy to use, especially over long periods of time, so they are a commonly used tool in the literature. There are also different ways of assessing environmental exposure. The majority of studies assess exposure to pollutants using questionnaires and biological samples. While questionnaires are a common method for describing environmental pollutant exposures, biological data from newborns (such as blood, urine, and meconium) are rarely used.

The present review will explore the effects of different chemical agents, including POPs, non‐POPs, and other environmental contaminants, on infant sleep in the first 1000 days of life. It will provide an overview of the existing evidence, identify toxic mechanisms that may impact sleep, and highlight gaps in the literature that require further investigation.

### 1.1 Persistent Organic Pollutants

Persistent organic pollutants (POPs) are carbon‐based chemicals. Due to their high stability, they have a long half‐life and are found throughout the environment. Most POPs are the result of human activities, such as the use of pesticides, industrial processes, or combustion. Once emitted, they can travel long distances through air or water and the food chain. Due to their persistence and liposolubility, POPs accumulate in the body through the food chain (Stockholm 2001).

Several categories of POPs have been examined for their potential effects on sleep. Polychlorinated biphenyls (PCBs) are organochlorine compounds formerly used in industrial applications. Although banned, their persistence in the environment and bioaccumulation in organisms remain a concern. Polychlorinated dibenzo‐*p*‐dioxins (PCDDs) and polychlorinated dibenzofurans (PCDFs) are toxic by‐products of industrial and combustion processes involving chlorine, classified amongst the 12 most hazardous pollutants, commonly referred to as the “dirty dozen” or dioxin‐like chemicals. Polybrominated diphenyl ethers (PBDEs) are flame retardants found in furniture, electronics, and textiles, accumulating in the body through ingestion and inhalation (Hooper and McDonald 2000). Per‐ and polyfluoroalkyl substances (PFAS), commonly known as “forever chemicals” are fluorinated compounds used in industrial and consumer products, including non‐stick cookware, food packaging, clothing, carpeting, and coatings (Houde et al. 2006). Finally, organochlorine pesticides (OCPs), such as DDT, hexachlorocyclohexanes (HCHs), aldrin, and chlordane, were extensively used in agriculture to control disease‐carrying insects (malaria and typhus) (Thompson et al. 2017), leading to widespread environmental contamination and toxic effects, particularly endocrine disruption.

**FIGURE 1:**
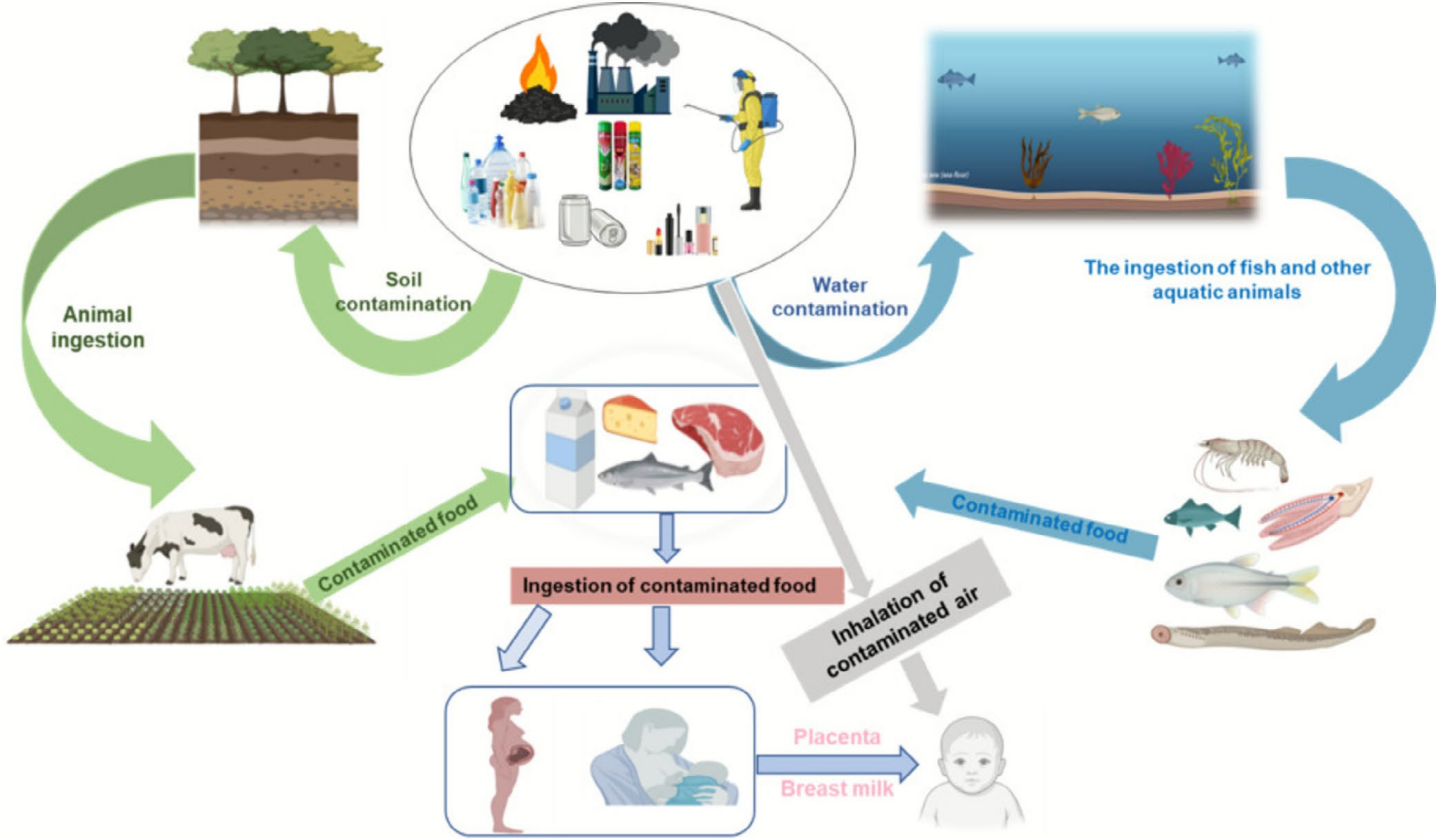
Release of persistent organic pollutants (POPs) into the environment and exposure ways.

The primary pathway of human exposure to POPs is through the ingestion of contaminated food, particularly animal products like eggs, fish, meat, and dairy products. However, exposure can also occur through inhalation and dermal absorption, especially for PBDEs. Maternal POPs can be transferred to offspring prenatally through the placenta (Vizcaino et al. 2014), and postnatally through breastfeeding (Grešner et al. 2021), and then bioaccumulate in foetal tissues, especially the brain (Figure 1). Their concentrations are commonly measured in blood (plasma/serum) and breast milk, reflecting their long‐term presence in the human body. This exposure can affect neurodevelopment as POPs are considered to be neurotoxic (Crépet et al. 2022).

POPs interfere with several biological systems essential for sleep regulation (Siegel 2004). Many of these compounds act as endocrine disruptors (PCBs, PBDEs, OCPs), altering hormonal balance, including thyroid hormones and melatonin, both of which regulate circadian rhythms (Green et al. 2021). These compounds also influence neurotransmitter systems, such as dopamine, serotonin, and GABA, potentially leading to sleep disturbances. In addition, POPs contribute to oxidative stress and neuroinflammation, which can affect sleep homeostasis. Some compounds, such as dioxins and PFAS, have been shown to alter circadian rhythms (Wang et al. 2023), further implicating them in sleep disturbances. Despite these known biological effects, direct evidence linking POPs exposure to sleep disturbances in children remains limited.

In their study Simeone et al. (2022) collected PCBs from maternal blood specimens at delivery and from children's blood at 6, 16, and 45 months. In this study, sleep was assessed through maternal reports of sleep problems, as well as feelings of anxiety and depression. The study found that exposure to PCB‐153 and PCB‐118 was associated with increased sleep problems, including disruptions in sleep duration and quality and increased anxiety‐related symptoms.

Ji et al. (2019) investigated the effects of prenatal PBDEs exposure on children's neurobehavioural development at ages 2 and 4 years in the general population of China, with a focus on sleep problems. PBDE levels were measured in umbilical cord blood samples taken immediately after birth. To assess sleep problems, they used the Child Behaviour Checklist (CBCL/1.5–5), which includes questions about sleep problems. According to the authors, higher prenatal PBDE levels were associated with sleep problems in girls, along with other problems such as somatic complaints, withdrawal, and internalising problems. In boys, sleep problems were not highlighted, but the exposure to PBDE was associated with somatic complaints and attention problems.

Epidemiological studies suggest that PFAS exposure is associated with metabolic dysfunction, impaired foetal growth, and neurodevelopmental disorders. In their study, Huang et al. (2023) explored the associations between prenatal PFAS exposure and infant sleep disturbances during the first year of life in a prospective urban cohort study. They recruited pregnant women from the Shanghai Birth Cohort (SBC), China and followed their children from birth to 12 months. Ten PFAS compounds were measured in blood serum collected in the first trimester. Sleep quality was measured using the Brief Infant Sleep Questionnaire. The study showed that prenatal PFAS exposure was also associated with longer sleep latency (β, 1.23; 95% CI, 0.08–2.38), more frequent nighttime awakenings (β, 0.04; 95% CI, 0.03–0.06), longer nighttime wakefulness (difference in night awakening times per quartile increase, 0.11; 95% CI, 0.05–0.18), snoring (RR = 1.79; 95% CI, 1.12–2.86), and earlier sleep onset in children aged 6–12 months. In this study, the authors did not assess whether the associations between prenatal PFAS exposure and infant sleep disturbances differed between boys and girls.

Similarly, in a recent cohort Xie et al. (2022), investigated the effects of prenatal PFAS exposure on children's neurobehavioural development at ages 2 and 4 years, with a focus on sleep problems. This study included 614 mother‐infant pairs from the Shanghai‐Minhang Birth Cohort Study (urban, China). Eight PFAS concentrations were measured in maternal plasma at 12–16 weeks of gestation. To assess sleep problems, they used the Child Behaviour Checklist (CBCL/1.5–5). Similar to the study on PBDEs, they have not directly investigated the association between PFAS exposure and sleep; they have examined overall neurobehavioural development, using sleep as a marker. According to the authors, higher prenatal PFAS levels were associated with sleep problems in girls. Unfortunately, the authors did not describe in detail those sleep problems.

For other POPs, such as dioxins (PCDDs, PCDFs), and OCPs, the literature on their effects on infant sleep remains limited. However, studies in adults suggest that exposure to dioxins is associated with severe insomnia symptoms, including difficulty initiating sleep, early awakenings, and difficulty maintaining sleep (Kondo et al. 2018), raising concerns about their potential impact on children's sleep regulation. Given their known endocrine‐disrupting and neurotoxic properties, dioxins may influence sleep regulation in children. Currently, no studies directly investigate the impact of OCPs on sleep in both adults and children. However, as endocrine disruptors, it is crucial to study this aspect, given their potential to interfere with hormones that regulate sleep–wake cycles. There is a notable gap in research on this topic, as studies have examined the effects of other pesticides (see below) on sleep but not organochlorines. This highlights the need for further research into the effect of dioxins and OCPs on sleep.

### 1.2 Non‐Persistent Pollutants

Non‐persistent pollutants are chemicals widely found in the environment and present in many everyday products, such as plastics, lubricants, solvents, plasticizers, and pesticides. Unlike POPs, non‐persistent pollutants do not remain in the environment for extended periods. These substances typically break down or dissipate more quickly, reducing their long‐term environmental impact (Campos and Freire 2016). Despite their shorter lifetime in the environment, low levels of exposure to these compounds can cause endocrine disorders. They can also act as neurotoxins, affecting the central nervous system and affecting neurotransmitters involved in sleep regulation, leading to sleep disturbances (Fabbri et al. 2023).

Several categories of non‐persistent pollutants have been examined for their potential impact on sleep disturbances. Phthalates are a group of endocrine‐disrupting chemicals commonly used as plasticizers in a wide range of consumer goods, including food packaging, personal care products, and household items (Heudorf et al. 2007). Pyrethroids are synthetic insecticides derived from natural pyrethrins (Chrustek et al. 2018) and are widely used in agriculture, household pest control, and disease vectors. In addition to their agricultural use, pyrethroids are essential in combating malaria (Accrombessi et al. 2023). Organophosphate pesticides (OPPs) are a class of neurotoxic compounds that have replaced organochlorine pesticides due to their lower environmental persistence but remain widely used in agriculture. They have numerous applications in clinical, household, and industrial settings (Terry 2012). Bisphenol A (BPA) is an industrial chemical used in the manufacture of polycarbonate plastics and epoxy resins, which are commonly found in food and drink packaging, receipt paper, and the lining of aluminium cans (Le et al. 2007).

Like POPs, the primary pathway of human exposure to non‐persistent pollutants is from the ingestion of contaminated food, and through inhalation and dermal contact. Infants are particularly vulnerable to exposure through prenatal transfer across the placenta and postnatal transfer via breastfeeding. Due to their rapid metabolism and elimination, non‐persistent pollutants are generally measured in biological fluids such as urine, blood (serum or plasma), saliva, breast milk, and amniotic fluid. Their frequent detection in urine samples highlights their widespread presence and continuous exposure in human populations.

Non‐persistent pollutants have been shown to disrupt endocrine function, alter neurotransmitter signalling, and induce oxidative stress, all of which may contribute to sleep disturbances. Phthalates interfere with multiple hormonal pathways, including thyroid, adrenal, and gonadal axes, and have been implicated in neurodevelopmental disorders (Braun et al. 2013). Pyrethroids can cross the blood–brain barrier and exert neurotoxic effects, potentially affecting sleep regulation (Costa 2015). Organophosphate pesticides inhibit acetylcholinesterase activity, leading to prolonged cholinergic stimulation in the nervous system, which may disrupt sleep–wake cycles (Furlong et al. 2006). BPA has been linked to alterations in stress response, mood disorders, and cardiometabolic dysfunction, which could indirectly impact sleep patterns (LaKind et al. 2014).

Despite the well‐documented biological effects, studies on the association between non‐persistent pollutants and sleep problems in children were limited. One study (Zhang et al. 2018) has examined the relationship between maternal bisphenol A exposure during early pregnancy and sleep problems amongst preschool children (3–6 years old). In this study, Zhang et al. included 1259 mother–child pairs from the Ma'anshan Cohort of the China‐Anhui Birth Cohort Study, an urban population from the industrial region of Anhui province. They collected PBA from maternal serum in the first trimester. In this study, sleep was assessed through maternal reports of sleep problems of their infants. The study showed that prenatal BPA exposure was strongly associated with sleep‐related problems (OR (odds ratio) = 1.44, 95% CI: 1.01–2.06) such as difficulty falling asleep, frequent night awakenings, and restless sleep. This association was found only in girls. In another cohort study, Geiger et al. (2023), investigated the effects of prenatal BPA exposure on children's neurobehavioural development amongst 2‐year‐olds, with a focus on sleep problems. This study involved 68 mother‐infant pairs from the Illinois Kids Development Study (USA, suburban/urban). BPA was quantified in pooled urine samples collected at five time points during pregnancy. To assess sleep problems, they used the Child Behaviour Checklist, which includes questions about sleep problems. Similar to other studies already mentioned, they have not directly investigated the association between BPA exposure and sleep; they have examined overall neurobehavioural development, using sleep as a marker. The study demonstrated that higher prenatal BPA levels were associated with sleep problems (OR = 2.33; 95% CI: 1.19–4.56; *p* = 0.013), such as difficulty falling asleep, night wakings, and decreased sleep duration. This association was found to be stronger in girls compared to boys.

Only one recent study (Huang et al. 2024), examined the impact of prenatal and postnatal phthalate exposure on the behavioural and emotional development of children aged 1.5–3 years in the general population of Taiwan, with a focus on sleep problems. The study followed 491 mother–child pairs, where phthalate levels were measured in maternal urine during the second trimester of pregnancy and in the urine of their children. To assess sleep problems, they used the Child Behaviour Checklist. According to the authors, higher prenatal dibutyl phthalate, metabolised as MnBP levels in maternal urine were significantly associated with an increased risk of sleep problems in children (OR = 41.34, 95% CI: 1.04–1632.84, *p* = 0.04), along with other problems such as anxiety and deficit hyperactivity disorder. No gender effects were researched.

To our knowledge, no direct studies have examined the effects of pyrethroids and organophosphate pesticides on sleep disturbances in children, but the well‐documented impact on neurodevelopment suggests a potential link. Pyrethroids have been shown to disrupt normal neural activity, potentially disturbing neurodevelopment by affecting the nervous system, which could influence indirectly the children's sleep patterns (Costa 2015). Organophosphate pesticides have been associated with cognitive deficits and attention disorders (Yolton et al. 2013), both of which may indirectly contribute to sleep disturbances. Given these biological mechanisms, further studies are necessary to investigate their potential impact on sleep in early life.

### 1.3 Air Pollution

The WHO defines air pollution as the contamination of the indoor or outdoor environment by any chemical, physical or biological agent that modifies the natural characteristics of the atmosphere. According to WHO statistics in 2023, around 99% of the world's population was living with poor air quality. The primary pathway of human exposure to air pollution is through the inhalation of contaminated air. Children are particularly vulnerable to air pollution because their lungs are not fully developed, and they have higher breathing rates relative to their body size (Bateson and Schwartz 2008).

Air pollution is linked to a wide range of infant health issues (Lin et al. 2023). It can contribute to respiratory diseases, such as chronic obstructive pulmonary disease, asthma, and obstructive sleep apnea, as well as non‐respiratory conditions like central nervous system diseases (Genc et al. 2012). Air pollution, which includes a mix of particulates and gases, can have both acute and chronic effects on children's sleep (Sánchez et al. 2019). The main air pollutants include particulate matter (PM1, PM2.5, PM10), polycyclic aromatic hydrocarbons (PAHs), and second‐hand smoke (SHS). Exposure to these pollutants can lead to sleep disturbances, reduced sleep duration, and poorer sleep quality in both adults and children (Liu et al. 2021).

Particulate matter (PM) is a complex mixture of solid particles and liquid droplets present in the environment (Zhang et al. 2021). It includes a variety of components, such as water, dust, acids, elemental carbon (black carbon), organic carbon, PAHs, metal dust, geographical mineral dust, and compounds like nitrates and sulphates.

PM is classified according to size, with coarse (PM10) particles being less than 10 μm in diameter, fine particles (PM2.5) including particles less than 2.5 μm, and ultrafine particles (PM1) being less than 0.1 μm. The health risks associated with PM increase as the particle size decreases, with smaller particles causing a greater risk to health.

PM can negatively impact children's sleep through several mechanisms. Inhalation of fine and ultrafine particles can cause inflammation and oxidative stress in the respiratory system (Leikauf et al. 2020), leading to sleep disturbances such as sleep apnea. Additionally, PM exposure can weaken the immune system, making children more susceptible to respiratory infections that disrupt sleep. These particles can also cross the blood–brain barrier (Elder et al. 2006), potentially affecting brain development and altering sleep–wake cycles. Furthermore, PM may induce epigenetic changes and endocrine effects (Shukla et al. 2019), contributing to long‐term sleep quality issues.

PM inhaled by the mother has been detected in the placenta and foetal organs. Additionally, evidence suggests that exposure to PM during pregnancy can impact foetal development (Guxens et al. 2018), predisposing children to sleep disturbances later in life. Although this exposure is indirect, the findings suggest that air pollution can have long‐lasting effects on newborn's sleep quality.

Unlike other chemical agents, the impact of PM on children's sleep is well‐studied in the literature.

In their nationwide study, Cai et al. (2023) selected a cohort of 115,023 preschool children (aged 3–7 years) from 551 Chinese cities (urban national cohort) to investigate the association between postnatal exposure to fine particulate matter (PM2.5) and sleep quality. Data were collected using the *Children's Sleep Habits Questionnaire*, with PM2.5 exposure estimated via a satellite‐based model. The study found significant associations between postnatal PM2.5 exposure and increased scores for sleep‐disordered breathing (SDB) (β, 0.03; 95% CI, 0.01–0.05) and daytime sleepiness (β, 0.25; 95% CI, 0.19–0.32). Specifically, PM2.5 exposure during the first 18 months showed stronger associations with sleep problems, including sleep duration (β, 0.03; 95% CI, 0.02–0.06), parasomnias (β, 0.05; 95% CI, 0.01–0.09), and excessive daytime sleepiness (β, 0.19; 95% CI, 0.14–0.24). This association was similar in both boys and girls.

Another study (An and Yu 2018) conducted in China examined the impact of PM2.5 on health amongst children and adolescents aged 5–17 years. Sleep duration was assessed using the Chinese version of the Pittsburgh Sleep Quality Index. In their study, An et al. observed a positive association between PM2.5 and excessive sleepiness (OR = 1.07; 95% CI = 1.04–1.11).

A study examined the impact of prenatal PM2.5 exposure on sleep in preschool‐aged children in Mexico City (urban). With PM2.5 exposure estimated using a satellite‐based model and child sleep measured via actigraphy at ages 4–5. The study found that exposure to PM2.5 during specific gestational windows affected sleep outcomes. Notably, exposure during weeks 31–35 of gestation was linked to decreased sleep duration, while exposure during weeks 1–8 was associated with reduced sleep efficiency in children. These findings suggest that prenatal PM2.5 exposure during sensitive periods of pregnancy can influence sleep patterns in children (Bose et al. 2019).

Two children's studies of air pollution have examined sleep‐disordered breathing (SDB), with heterogeneous results. In their study, Gokdemir et al. (2021) selected a random sample of primary school students in Turkey (5–13 years) from both urban and rural areas. Data were collected using the Paediatric Sleep Questionnaire, asthma, and allergic rhinitis questionnaires, completed by the parents. The study found a high prevalence of SDB symptoms in children living in both urban and rural areas. However, Accinelli et al. (2015) conducted a study on children aged 9–15 residing at high altitudes and exposed to traditional biomass‐fuelled stoves. Using the same questionnaires, they reported no findings of SDB in these children.

One cross‐sectional study in Iran has examined the effect of air pollution on children's sleep. They distributed 6000 questionnaires to children aged 6–12 attending public schools in five distinct neighbourhoods, selected based on air quality measures. This study found that children exposed to high levels of pollutants were more likely to habitually snore (risk ratio = 1.87; 95% CI: 1.38–2.47; *p* < 0.001), thus disrupting their sleep quality (Kheirandish‐Gozal et al. 2014).

Polycyclic aromatic hydrocarbons (PAHs) are widespread environmental pollutants, predominantly resulting from the incomplete combustion and pyrolysis of organic substances. These sources include tobacco, petroleum products, fossil fuels, and even certain food preparation methods. Their persistence in the atmosphere raises concerns about their potential toxic effects on human health after prolonged exposure (Mallah et al. 2022). PAHs are ubiquitous and can enter the human body by inhalation, ingestion, and skin contact. Most studies have measured PAH concentrations in urine (Jacob and Seidel 2002), blood (Singh et al. 2008), including plasma/serum (Mlyczyńska et al. 2023), and even saliva (Stéphan‐Blanchard et al. 2016) to assess tobacco exposure. PAHs can cross the placenta and expose the foetus prenatally (Paquette et al. 2023).

PAHs disrupt the endocrine system and neurological development, which affects sleep–wake cycles. While the exact mechanisms by which PAHs influence sleep disorders remain unclear, toxicological studies have demonstrated that exposure to PAHs, specifically benzo[a]pyrene, can interfere with DNA repair mechanisms and disturb circadian rhythms (Yin et al. 2020). This suggests that PAHs may impact sleep by altering circadian rhythms and reducing melatonin secretion, potentially leading to sleep disorders.

Unfortunately, to date, no studies have investigated the potential association between PAHs exposure and children's sleep. However, a study has examined this exposure in adults and found an association (Zhao et al. 2024).

Another critical source of indoor air pollution is SHS, which shares harmful effects on children's health and sleep. Smoking is responsible for over 7 million deaths annually worldwide. More than 85% of these fatalities are due to direct smoking, and around 0.89 million are attributed to SHS exposure (World Health Organization 2018). According to the WHO, SHS, also known as passive smoke, environmental tobacco smoke, or involuntary smoke, is a combination of sidestream smoke emitted from the burning end of a cigarette or other smoked tobacco product and mainstream smoke exhaled by a smoker and mixed with the surrounding air (World Health Organization 1949).

SHS can occur in a variety of indoor and outdoor settings, including the home, public spaces, and enclosed environments, making children particularly vulnerable.

Children's susceptibility to SHS results from their higher breathing rates, smaller lung size, and developing immune systems. SHS exposure has been linked to severe respiratory issues, such as asthma (Vork et al. 2007), respiratory infections (Continente et al. 2019), and reduced lung function (Arghir et al. 2013). Research has also identified a strong link between SHS exposure and sleep disturbances in children.

Two recent sleep reviews, Wallace et al. (2023), and Liu et al. (2021), have included studies reporting positive associations between SHS exposure and sleep. These reviews examined sleep in both adults and children. In this section, we will present only the studies related to children's sleep described in these reviews and other recent studies that have been done since.

Yolton et al. (2010) conducted a study in the United States, exploring the relationship between SHS exposure and sleep patterns in children (6–12 years) with asthma, using the Children's Sleep Habits Questionnaire to assess sleep. The cohort was composed of urban, low‐ to middle‐income families, a population particularly vulnerable to indoor air pollution. They found that SHS exposure, as measured by serum cotinine levels, was associated with longer sleep onset delays (OR = 1.53, 95% CI: 1.15–2.03, *p* = 0.004), SDB (OR = 1.26; 95% CI: 1.04–1.52; *p* = 0.02), parasomnias (OR = 1.95; 95% CI: 1.36–2.79; *p* = 0.0002), daytime sleepiness (OR = 0.33; 95% CI: 0.05–0.61; *p* = 0.022), and general sleep disturbances in these infants.

Jara et al. (2015) conducted a literature review on SHS exposure and SDB in children (3–11 years). The review confirmed a strong association between SHS exposure and habitual snoring, OSA, and sleep‐related hypoxia, highlighting the adverse respiratory effects of SHS on sleep.

In a recent US study, Tsou et al. (2024), explored the relationship between environmental tobacco smoke (SHS) exposure and sleep habits in children (aged 3–12.9 years) with asthma and mild sleep‐disordered breathing. Using polysomnographic measurements and specific questionnaires (*Paediatric Sleep Questionnaire‐Sleep‐Related Breathing Disorder scale*), SDB‐specific quality of life (OSA‐18), sleepiness (modified Epworth Sleepiness Score), researchers observed that SHS exposure was associated with increased SDB symptoms, decreased quality of life, and a slight increase in arousal index. These results highlight the negative impact of SHS on the sleep of asthmatic children.

In their study, Groner et al. (2019) investigated the relationship between SHS exposure and SDB in children aged 2–5 years in the United States. SHS exposure was determined by hair nicotine levels. Sleep was assessed through parental reports. The study found an association between objectively measured SHS exposure and sleep‐related breathing problems in this preliminary cross‐sectional study of healthy toddlers.

Wafula et al. (2025) conducted a cross‐sectional study in an urban informal settlement in Kampala, Uganda, examining the association between SHS exposure in children and their sleep outcomes. They assessed SDB in children aged 6–59 months through parent‐reported questionnaires. They found that exposure to cigarette‐related compounds during pregnancy (through maternal active smoking) or exposure to SHS in the first 6 months of life was associated with an increased risk of SDB (Prevalence Ratio (PR) = 1.78, 95% CI: 1.21–2.61). The risk remained significant when exposure occurred during both periods (PR = 1.48, 95% CI: 1.02–2.13).

In a recent study, Ramírez Benítez et al. (2025) examined how SHS exposure affects preschool children's sleep quality (aged 3–7 years) in Cuba using medical records collected between 2015 and 2018. The study found that both continuous (five to seven times per week) and transient (two to three times per week) exposure to household tobacco smoke significantly increased sleep restlessness. Despite all children getting at least 7 h of sleep per night, those exposed to smoke experienced greater disturbances. Boys showed higher levels of sleep restlessness compared to girls (Ramírez Benítez et al. 2025). These findings highlight the negative impact of household smoke exposure on children's sleep, particularly in families with a middle socioeconomic status (Ramírez Benítez et al. 2025), where environmental conditions and household routines may exacerbate vulnerability, leading to increased sleep restlessness and disturbances despite an adequate sleep duration.

### 1.4 Waters Contaminants

Water pollution is an increasing global concern, with various contaminants affecting human health. While much research has focused on their effects on metabolism, endocrine function, and neurological health, their potential impact on sleep, particularly in children, remains under‐investigated.

Some pollutants, such as nitrates, perchlorates, pharmaceutical residues, and microplastics, may disrupt sleep by interfering with thyroid function, neurotransmitter activity, or respiratory health. However, these contaminants are increasingly present in our environment, but their impact on children's sleep has not been studied.

Nitrate is a naturally occurring anion and a key part of the nitrogen cycle, absorbed by plants for protein synthesis. It's commonly found in vegetables, grains, agricultural fertilisers, and preserved meats (Crawford 1995). However, increased fertiliser use has significantly raised nitrate levels in water. Humans are exposed to nitrate through contaminated water and food. Nitrate disrupts thyroid function by interfering with iodine uptake, potentially leading to thyroid disorders. In the body, nitrate converts to nitrite, forming N‐nitroso compounds, known carcinogens. High nitrate consumption is linked to cancer, reproductive issues, and impaired thyroid function (Serrano‐Nascimento and Nunes 2022). Additionally, nitrate could disrupt sleep by interfering with thyroid function, leading to hormone imbalances that affect sleep quality and duration. There is no study specifically examining this effect in children.

Perchlorates, commonly found in rocket propellants, fireworks, and some fertilisers, can contaminate water supplies and disrupt thyroid function by interfering with iodine uptake (Leung et al. 2014). This disruption is relevant because thyroid hormones are critical for growth, development, and brain function in children. While there isn't extensive research directly linking perchlorates to sleep disturbances in children, the potential impact on thyroid function (Tonacchera et al. 2004) suggests that it could indirectly affect sleep, given the role of thyroid hormones in regulating sleep patterns.

Pharmaceutical residues enter water bodies through various pathways, including improper disposal of medications, excretion by humans and animals, and agricultural runoff (Sharma et al. 2022). These compounds are highly stable, slow to biodegrade, and tend to accumulate in fatty tissues. Although they primarily impact aquatic life and may pose risks to human health if they contaminate drinking water (Patel et al. 2019). While research into the effects of pharmaceutical residues on sleep is not developed, their presence in water raises concerns about their impact on sleep.

Microplastics are tiny plastic fragments resulting from plastic waste generated by human activities. These microscopic plastics are discharged into the environment and eventually end up in aquatic environments, including seawater, wastewater, and freshwater (Kye et al. 2023). They could re‐enter the human body through food ingestion, including seafood, commercial processed fish, sea salt, honey, and beer (Yang et al. 2015). Their spread in the environment is influenced by their properties, such as density, hydrophobicity, and persistence. Emerging research suggests that microplastics may have various adverse health effects (Lee et al. 2023), including neurotoxicity (Prüst et al. 2020). Microplastics can disrupt the central nervous system and may interfere with neurotransmitters that regulate sleep, though more research is needed to confirm these speculations.

Finally, only a few studies have examined the relationship between water contaminants and children's sleep. One study by Doran and Aschengrau (2022) investigated the long‐term neurotoxic effects of early‐life exposure to tetrachloroethylene (PCE) through contaminated drinking water in Cape Cod, Massachusetts. Using data from the Cape Cod Health Study, they estimated PCE exposure with a validated leaching and transport model. Sleep quality was assessed through self‐administered questionnaires. The results suggest that early‐life exposure to PCE is associated with a moderate increase in the risk of reporting breathing pauses during sleep in adulthood (OR = 1.57, 95% CI: 0.92–2.68).

While direct evidence linking water contaminants to sleep disturbances in children is not developed, the potential health risks associated with these pollutants highlight the need for further research.

## 2 Discussion

The studies in our review clearly show that persistent organic pollutants may cause risks to children's sleep regulation. While studies on PCBs, PBDEs, and PFAS have shown associations with sleep problems in children, research remains limited, especially for other POPs like dioxins and OCPs. Given children's vulnerability to environmental toxins and the potential long‐term consequences of sleep disturbances in early life, future research should focus on filling these knowledge gaps and investigating the impact of less‐studied pollutants like dioxins and OCPs on sleep.

Non‐persistent pollutants, including pyrethroids, OPPs, BPA, and phthalates may break down more rapidly in the environment compared to POPs, but they still have significant risks to child neurodevelopment, impacting sleep regulation. Pyrethroids and OPPs, which have strong links to neurodevelopmental issues, have limited studies specifically associating them with sleep problems in children, although effects in adolescents suggest potential risks. Zamora et al. (2021), indicate that maternal exposure to chlorpyrifos during pregnancy was associated with longer sleep duration and later sleep timing in adolescents, with stronger effects observed in female offspring.

As pyrethroids or OPPs, phthalates may be linked to sleep disturbances in adolescents, but no studies have assessed this association in children. In their study, Sears et al. indicate that phthalates are associated with shorter sleep duration in adolescents (Sears and Braun 2021), whereas Zamora et al. (2022) found that higher exposure to 14 phthalate metabolite levels may be associated with longer sleep duration and later sleep timing amongst adolescent offspring, which varied by gender. These findings would raise concerns that similar mechanisms may affect younger populations.

Only, BPA, another non‐persistent pollutant, has been more directly linked to sleep disturbances in children, including difficulty falling asleep and increased nighttime awakenings, particularly in girls, based on prenatal exposure studies (Zhang et al. 2018).

An important aspect observed in existing studies would be the potential gender‐specific effects of POPs and non‐persistent pollutants on sleep. Research in children would suggest that sleep disturbances related to PBDEs, PFAS, phthalates, and BPA exposure would appear more pronounced in girls than in boys. This difference could be attributed to the endocrine‐disrupting properties of these chemicals, which interfere with sex hormone regulation. Given the role of oestrogen and other hormones in modulating the sleep–wake cycle, it would be plausible that endocrine disruption leads to sex‐specific vulnerabilities in sleep regulation. Sex differences in sleep emerge very early in life and persist throughout childhood and adulthood. As highlighted by Franco et al. (2020), these differences are largely mediated by oestradiol, which influences cortical maturation and brain function from the foetal stage. Such hormonal modulation may explain why girls appear more vulnerable to sleep disturbances associated with environmental exposures. Future studies should explore whether these patterns would extend to children and investigate the mechanisms underlying these gender differences.

Air pollution, including particulate matter (PM) and secondhand smoke (SHS), would also be associated with sleep disturbances in children. Due to their underdeveloped respiratory systems, children would be particularly vulnerable to airborne pollutants, which would contribute to sleep‐disordered breathing (SDB), obstructive sleep apnea (OSA), and poor sleep quality. Fine particulate matter, such as PM2.5 and PM1, would have been linked to respiratory issues that disrupt sleep. SHS exposure would also be associated with SDB, OSA, and snoring. While PM and SHS would have been extensively studied, fewer investigations would have explored the effects of Polycyclic Aromatic Hydrocarbons (PAHs), which are highly prevalent in industrial environments, on sleep in children, leaving critical knowledge gaps. A study conducted by Zhao et al. (2024) examined NHANES data from 7730 adults and found associations between urinary PAH metabolites and sleep disturbances.

There are also concerns about other emerging chemical pollutants such as nitrates, perchlorates, pharmaceutical residues, and microplastics in water. New data suggest a possible link between water contaminants and sleep disturbances in children (Doran and Aschengrau 2022). Prenatal and early childhood exposure to tetrachloroethylene in drinking water has been associated with adverse sleep outcomes in adulthood.

Most of the studies rely on self‐reported sleep outcomes and lack objective measures such as polysomnography (PSG) or actigraphy. Self‐reports are susceptible to recall bias and subjective interpretation, potentially compromising the accuracy of the data collected. Actigraphy provides a non‐invasive, continuous measure of sleep patterns by tracking movement, offering more objective and reliable data. PSG provides comprehensive insights into sleep architecture by measuring brain activity, eye movements, muscle activity, and breathing patterns. Investigating sleep specifically within this context provides valuable insights into the maturation of the nervous system. Future research should prioritise objective sleep measurements, such as actigraphy and PSG, to enhance accuracy and reliability. Current studies most often present sleep as an indicator of neurodevelopment in their results, rather than studying it directly. Additionally, the measurement of environmental exposure in children has been indirect, as most studies have focused on maternal exposure rather than direct assessments through urine, blood, or meconium samples.

Our objective was to emphasise the lack of clinical data concerning human newborns. Consequently, we restricted our review to human studies in order to highlight this gap. While some experimental studies exist, they remain relatively scarce and heterogeneous. Direct extrapolation from animal models to infants is challenging due to differences in exposure levels, developmental stages, and species‐specific physiology. This methodological choice allowed us to underline the urgent need for more human‐based research in this area, but it also represents a limitation of our review.

It is also important to consider the role of co‐exposures to chemical mixtures and co‐exposure to physical agents. Children are often exposed to complex mixtures of chemicals, yet most studies examine pollutants in isolation. Understanding the cumulative effects of co‐exposure is critical for a comprehensive assessment of how environmental factors influence sleep. Given that endocrine disruptors, neurotoxic chemicals, and respiratory pollutants may interact in ways that amplify their adverse effects, future research should incorporate multi‐exposure models to capture these interactions.

Beyond chemical exposures, social determinants such as socioeconomic status (Papadopoulos and Sosso 2023), maternal education, and area deprivation are known to influence sleep health (Barazzetta and Ghislandi 2016). However, most of the studies included in this review did not examine socioeconomic factors such as income, poverty, or neighbourhood deprivation in relation to sleep outcomes. The absence of these variables means that potential confounding effects were not considered, which may introduce bias into the associations between chemical exposures and sleep outcomes. Disadvantaged families are both more exposed to pollutants and more vulnerable to sleep disturbances, underscoring the importance of systematically including socioeconomic measures in future research. This highlights an important methodological gap that should be addressed in upcoming studies.

In addition, several studies emphasise that poverty and structural disinvestment increase both exposure to pollutants and vulnerability to sleep disturbances. Communities experiencing social disadvantage are more likely to reside near emission sources, in housing with poor indoor air quality, and to be subject to chronic stressors that disrupt sleep regulation. Billings et al. (2020), provide evidence that both the physical environment (e.g., noise, air quality, housing conditions) and the social environment (e.g., socioeconomic status, neighbourhood safety, discrimination) are closely linked to sleep health and disorders. Their review highlights that sleep disparities cannot be understood without considering these contextual determinants. Payne‐Sturges et al. (2023), document disparities in toxic chemical exposures and associated neurodevelopmental outcomes, showing that minority and low‐income populations bear a disproportionate burden due to systemic inequities in housing, neighbourhood conditions, and access to resources. This scoping review and evidence map underscores how cumulative exposures and social disadvantage interact to affect child health outcomes. Hoyniak et al. (2024), demonstrate that social disadvantage during pregnancy is associated with altered maternal sleep and circadian rhythms, which in turn are related to differences in neonatal brain development. Their findings suggest that inequities in sleep and exposure begin even before birth, with potential long‐term consequences for neurodevelopment. Therefore, poverty and social disadvantage should be considered as co‐exposures when examining the impact of pollutants on sleep. Future studies should also integrate socioeconomic measures to avoid underestimating the role of structural inequities. Together, these studies highlight that the socioeconomic context is not only a confounding factor but also a potential modifier of pollutant–sleep associations, reinforcing the need for an integrative approach that jointly considers chemical exposures, social determinants, and their cumulative effects on child sleep and development.

In summary, future studies should address research gaps, particularly concerning less‐studied pollutants, and adopt more objective methods for assessing sleep. Future studies should prioritise studies on infants and young children rather than extrapolating findings from adults. More studies should analyze gender differences, as existing evidence suggests that girls may be more vulnerable to sleep disturbances caused by endocrine disruptors. Instead of studying pollutants in isolation, research should explore the combined effects of multiple exposures, including those from air, water, and food.

In France, prevention programmes such as the FEES initiative (Femmes Enceintes Environnement et Santé) (FEES 2023), and the governmental “1000 days” programme provide guidance to families, focusing on pregnant women and infants. These programmes emphasise practical measures to reduce environmental exposures during this critical developmental period. In addition to these initiatives, parents can adopt simple strategies in their daily routines, such as regularly ventilating the home, reducing exposure to tobacco smoke, and limiting contact with products containing endocrine disruptors. Such measures provide immediate and effective ways to minimise environmental risks during this highly sensitive development period.

With the principle of precaution, stricter regulations on POPs and endocrine disruptors should be implemented, particularly in consumer products such as plastics, cosmetics, and pesticides. Pregnant women should be protected from air pollution and second‐hand smoke exposure through urban pollution control policies and public health campaigns. Water quality should be monitored and improved, with efforts to reduce contaminants such as nitrates, perchlorates, and pharmaceutical residues in drinking water.

Finally, paediatricians and perinatal healthcare professionals should receive training on the impact of environmental pollutants on infant sleep. Beyond family counselling during prenatal and postnatal consultations, paediatricians and midwives can play a key role in raising awareness about environmental risks and promoting strategies to reduce exposures. They are involved at both the individual and institutional levels: at an individual level, they provide counselling during prenatal and postnatal consultations; at an institutional level, they advocate for safer practises in neonatal care units. Recent studies have shown that infants in intensive care units may be exposed to phthalates, bisphenol A, parabens (Iribarne‐Durán et al. 2019), volatile organic compounds (El‐Metwally et al. 2018), and ethanol excipients from medications (Stefanak et al. 2020), underscoring the need for vigilance and preventive measures.

Public awareness campaigns should educate parents on practical ways to reduce exposure, such as choosing BPA‐free products, avoiding heated plastics, opting for organic foods, and filtering tap water. Public health policies should focus on improving indoor and outdoor air quality, particularly in schools.

## 3 Conclusions

This review highlights the methods and results of the previous studies conducted to study the significant impact of environmental chemical exposure on children's sleep.

POPs, non‐persistent pollutants, air pollution, and SHS are all associated with disruptions in sleep's quality and duration amongst infants and children. Despite the extensive research on adults, there remains a notable gap in our understanding of how environmental pollutants affect children's sleep. This lack of studies focusing on children has resulted in limited associations in the existing literature.

Although animal studies have been conducted, we chose to focus on research involving human children, where potential effects, particularly in girls, appear to be more pronounced. This gap in research is concerning, especially given that children are more vulnerable to environmental pollutants due to their developmental stages. It would be relevant to study other contaminants, particularly those present in water, that are suspected to have neurotoxic properties, such as microplastics and nitrates.

Future research should be designed to thoroughly assess these exposures and their effects on sleep, using more rigorous methods specifically designed for sleep and considering potential confounding factors. Policies aimed at reducing children's exposure to these harmful environmental agents are urgently needed to protect their health and contribute to better sleep quality.

To conclude, based on the known mechanisms of action of environmental pollutants, it is possible to hypothesise that these pollutants may impact children's sleep, but current knowledge remains limited. Addressing the gaps in research, particularly concerning less‐studied pollutants, and adopting more objective methods for assessing sleep will be essential to have a better understanding of this association and its long‐term effect. Additionally, it would be interesting to study co‐exposures to chemical mixtures and co‐exposure to physical agents (ambient noise, light, ionising and non‐ionising radiation, magnetic field, ambient temperature).

## Author Contributions

**Zeina Halbouty:** conceptualization, methodology, investigation, resources, formal analysis, writing – original draft, visualization, and project administration. **Debora Tuka:** validation, writing – review and editing. **Erwan Stephan‐Blanchard:** conceptualization, supervision, writing – review and editing. **Veronique Bach:** writing – review and editing. **Pierre Tourneux:** writing – review and editing. **Elodie Haraux:** writing – review and editing. **Karen Chardon:** conceptualization, supervision, writing – review and editing, and validation.

## Funding

The authors have nothing to report.

## Conflicts of Interest

The authors declare no conflicts of interest.

## Data Availability

Data sharing not applicable to this article as no datasets were generated or analysed during the current study.

## References

[jsr70286-bib-0001] Accinelli, R. A. , O. Llanos , L. M. López , et al. 2015. “Caregiver Perception of Sleep‐Disordered Breathing‐Associated Symptoms in Children of Rural Andean Communities Above 4000 Masl With Chronic Exposure to Biomass Fuel.” Sleep Medicine 16, no. 6: 723–728. 10.1016/j.sleep.2015.02.536. 26002760

[jsr70286-bib-0002] Accrombessi, M. , J. Cook , E. Dangbenon , et al. 2023. “Efficacy of Pyriproxyfen‐Pyrethroid Long‐Lasting Insecticidal Nets (LLINs) and Chlorfenapyr‐Pyrethroid LLINs Compared With Pyrethroid‐Only LLINs for Malaria Control in Benin: A Cluster‐Randomised, Superiority Trial.” Lancet 401, no. 10375: 435–446. 10.1016/S0140-6736(22)02319-4. 36706778

[jsr70286-bib-0003] An, R. , and H. Yu . 2018. “Impact of Ambient Fine Particulate Matter Air Pollution on Health Behaviors: A Longitudinal Study of University Students in Beijing, China.” Public Health 159: 107–115. 10.1016/j.puhe.2018.02.007. 29567011

[jsr70286-bib-0004] Ardura, J. , J. Andrés , J. Aldana , and M. A. Revilla . 1995. “Development of Sleep‐Wakefulness Rhythm in Premature Babies.” Acta Paediatrica 84, no. 5: 484–489. 10.1111/j.1651-2227.1995.tb13679.x. 7633140

[jsr70286-bib-0005] Arghir, O. C. , E. Danteş , R. Stoicescu , et al. 2013. “Parental Environmental Tobacco Smoking and the Prevalence of Respiratory Diseases in Primary School Children.” Pneumologia 62, no. 3: 178–181. 24274004

[jsr70286-bib-0006] Barazzetta, M. , and S. Ghislandi . 2016. “Family Income and Material Deprivation: Do They Matter for Sleep Quality and Quantity in Early Life? Evidence From a Longitudinal Study.” Sleep 40, no. 3: zsw066. 10.1093/sleep/zsw066. PMC6410939 28364413

[jsr70286-bib-0093] Bateson, T. F. , and J. Schwartz . 2008. “Children's Response to Air Pollutants.” Journal of Toxicology and Environmental Health, Part A 71, no. 3: 238–243. 10.1080/15287390701598234. 18097949

[jsr70286-bib-0007] Bathory, E. , and S. Tomopoulos . 2017. “Sleep Regulation, Physiology and Development, Sleep Duration and Patterns, and Sleep Hygiene in Infants, Toddlers, and Preschool‐Age Children.” Current Problems in Pediatric and Adolescent Health Care 47, no. 2: 29–42. 10.1016/j.cppeds.2016.12.001. 28117135

[jsr70286-bib-0008] Billings, M. E. , L. Hale , and D. A. Johnson . 2020. “Physical and Social Environment Relationship With Sleep Health and Disorders.” Chest 157, no. 5: 1304–1312. 10.1016/j.chest.2019.12.002. 31870910 PMC7268445

[jsr70286-bib-0009] Boekelheide, K. , B. Blumberg , R. E. Chapin , et al. 2012. “Predicting Later‐Life Outcomes of Early‐Life Exposures.” Environmental Health Perspectives 120, no. 10: 1353–1361. 10.1289/ehp.1204934. 22672778 PMC3491941

[jsr70286-bib-0010] Bose, S. , K. R. Ross , M. J. Rosa , et al. 2019. “Prenatal Particulate Air Pollution Exposure and Sleep Disruption in Preschoolers: Windows of Susceptibility.” Environment International 124: 329–335. 10.1016/j.envint.2019.01.012. 30660846 PMC6615028

[jsr70286-bib-0011] Braun, J. M. , S. Sathyanarayana , and R. Hauser . 2013. “Phthalate Exposure and Children's Health.” Current Opinion in Pediatrics 25, no. 2: 247–254. 10.1097/MOP.0b013e32835e1eb6. 23429708 PMC3747651

[jsr70286-bib-0012] Cai, J. , Y. Shen , Y. Zhao , et al. 2023. “Early‐Life Exposure to PM2.5 and Sleep Disturbances in Preschoolers From 551 Cities of China.” American Journal of Respiratory and Critical Care Medicine 207, no. 5: 602–612. 10.1164/rccm.202204-0740OC. 36170612

[jsr70286-bib-0013] Campos, É. , and C. Freire . 2016. “Exposure to Non‐Persistent Pesticides and Thyroid Function: A Systematic Review of Epidemiological Evidence.” International Journal of Hygiene and Environmental Health 219, no. 6: 481–497. 10.1016/j.ijheh.2016.05.006. 27265299

[jsr70286-bib-0014] Chaput, J. P. , C. E. Gray , V. J. Poitras , et al. 2016. “Systematic Review of the Relationships Between Sleep Duration and Health Indicators in School‐Aged Children and Youth.” Applied Physiology, Nutrition, and Metabolism 41, no. 6: S266–S282. 10.1139/apnm-2015-0627. 27306433

[jsr70286-bib-0015] Chrustek, A. , I. Hołyńska‐Iwan , I. Dziembowska , et al. 2018. “Current Research on the Safety of Pyrethroids Used as Insecticides.” Medicina 54, no. 4: 61. 10.3390/medicina54040061. 30344292 PMC6174339

[jsr70286-bib-0016] Continente, X. , T. Arechavala , E. Fernàndez , et al. 2019. “Burden of Respiratory Disease Attributable to Secondhand Smoke Exposure at Home in Children in Spain (2015).” Preventive Medicine 123: 34–40. 10.1016/j.ypmed.2019.02.028. 30817956

[jsr70286-bib-0017] Costa, L. G. 2015. “Chapter 9—The Neurotoxicity of Organochlorine and Pyrethroid Pesticides.” In Handbook of Clinical Neurology, edited by M. Lotti and M. L. Bleecker , 135–148. Elsevier. 10.1016/B978-0-444-62627-1.00009-3 26563787

[jsr70286-bib-0018] Crawford, N. M. 1995. “Nitrate: Nutrient and Signal for Plant Growth.” Plant Cell 7, no. 7: 859–868. 10.1105/tpc.7.7.859. 7640524 PMC160877

[jsr70286-bib-0019] Crépet, A. , P. Vasseur , J. Jean , et al. 2022. “Integrating Selection and Risk Assessment of Chemical Mixtures: A Novel Approach Applied to a Breast Milk Survey.” Environmental Health Perspectives 130, no. 3: 035001. 10.1289/EHP8262. 35238606 PMC8893236

[jsr70286-bib-0020] Davis, K. F. , K. P. Parker , and G. L. Montgomery . 2004. “Sleep in Infants and Young Children: Part Two: Common Sleep Problems.” Journal of Pediatric Health Care 18, no. 3: 130–137. 10.1016/S0891-5245(03)00150-0. 15129213

[jsr70286-bib-0021] Deak, M. C. , and R. Stickgold . 2010. “Sleep and Cognition.” Wiley Interdisciplinary Reviews: Cognitive Science 1, no. 4: 491–500. 10.1002/wcs.52. 26271496 PMC5831725

[jsr70286-bib-0022] Doran, C. R. , and A. Aschengrau . 2022. “Prenatal and Early Childhood Exposure to Tetrachloroethylene (PCE)‐Contaminated Drinking Water and Sleep Quality in Adulthood: A Retrospective Cohort Study.” Environmental Health 21, no. 1: 15. 10.1186/s12940-021-00819-7. 35033085 PMC8760772

[jsr70286-bib-0023] Elder, A. , R. Gelein , V. Silva , et al. 2006. “Translocation of Inhaled Ultrafine Manganese Oxide Particles to the Central Nervous System.” Environmental Health Perspectives 114, no. 8: 1172–1178. 10.1289/ehp.9030. 16882521 PMC1552007

[jsr70286-bib-0024] El‐Metwally, D. , K. Chain , M. P. Stefanak , et al. 2018. “Urinary Metabolites of Volatile Organic Compounds of Infants in the Neonatal Intensive Care Unit.” Pediatric Research 83, no. 6: 1158–1164. 10.1038/pr.2018.52. 29768398 PMC6504844

[jsr70286-bib-0025] Fabbri, L. , R. Garlantézec , K. Audouze , et al. 2023. “Childhood Exposure to Non‐Persistent Endocrine Disrupting Chemicals and Multi‐Omic Profiles: A Panel Study.” Environment International 173: 107856. 10.1016/j.envint.2023.107856. 36867994

[jsr70286-bib-0026] Fagioli, I. , and P. Salzarulo . 1982. “Temporal Organization of Sleep Cycles in Infants Over 24‐Hour Periods.” Revue d'Électroencéphalographie et de Neurophysiologie Clinique 12, no. 4: 344–348. 10.1016/s0370-4475(82)80024-5. 7170380

[jsr70286-bib-0027] FEES . 2023. Projet FEES—Femmes Enceintes Environnement et Santé.

[jsr70286-bib-0028] Franco, P. , B. Putois , A. Guyon , et al. 2020. “Sleep During Development: Sex and Gender Differences.” Sleep Medicine Reviews 51: 101276. 10.1016/j.smrv.2020.101276. 32109833

[jsr70286-bib-0029] Furlong, C. E. , N. Holland , R. J. Richter , A. Bradman , A. Ho , and B. Eskenazi . 2006. “PON1 Status of Farmworker Mothers and Children as a Predictor of Organophosphate Sensitivity.” Pharmacogenetics and Genomics 16, no. 3: 183–190. 10.1097/01.fpc.0000189796.21770.d3. 16495777

[jsr70286-bib-0030] Geiger, S. D. , S. Musaad , J. Hill , A. Aguiar , and S. Schantz . 2023. “Sex‐Specific Associations Between Urinary Bisphenols Concentrations During Pregnancy and Problematic Child Behaviors at Age 2 Years.” Neurotoxicology and Teratology 96: 107152. 10.1016/j.ntt.2023.107152. 36642394 PMC10170945

[jsr70286-bib-0031] Genc, S. , Z. Zadeoglulari , S. H. Fuss , and K. Genc . 2012. “The Adverse Effects of Air Pollution on the Nervous System.” Journal of Toxicology 2012: 782462. 10.1155/2012/782462. 22523490 PMC3317189

[jsr70286-bib-0032] Gertner, S. , C. W. Greenbaum , A. Sadeh , Z. Dolfin , L. Sirota , and Y. Ben‐Nun . 2002. “Sleep–Wake Patterns in Preterm Infants and 6 Month's Home Environment: Implications for Early Cognitive Development.” Early Human Development 68, no. 2: 93–102. 10.1016/S0378-3782(02)00018-X. 12113995

[jsr70286-bib-0033] Giaginis, C. , S. Theocharis , and A. Tsantili‐Kakoulidou . 2012. “Current Toxicological Aspects on Drug and Chemical Transport and Metabolism Across the Human Placental Barrier.” Expert Opinion on Drug Metabolism & Toxicology 8, no. 10: 1263–1275. 10.1517/17425255.2012.699041. 22780574

[jsr70286-bib-0034] Gokdemir, Y. , E. Civelek , B. Cakir , et al. 2021. “Prevalence of Sleep‐Disordered Breathing and Associated Risk Factors in Primary School Children in Urban and Rural Environments.” Sleep & Breathing 25, no. 2: 915–922. 10.1007/s11325-020-02206-x. 33030645

[jsr70286-bib-0035] Grandjean, P. , and P. J. Landrigan . 2014. “Neurobehavioural Effects of Developmental Toxicity.” Lancet Neurology 13, no. 3: 330–338. 10.1016/S1474-4422(13)70278-3. 24556010 PMC4418502

[jsr70286-bib-0036] Green, M. E. , V. Bernet , and J. Cheung . 2021. “Thyroid Dysfunction and Sleep Disorders.” Frontiers in Endocrinology 12: 725829. 10.3389/fendo.2021.725829. 34504473 PMC8423342

[jsr70286-bib-0037] Grešner, P. , M. Zieliński , D. Ligocka , K. Polańska , W. Wąsowicz , and J. Gromadzińska . 2021. “Environmental Exposure to Persistent Organic Pollutants Measured in Breast Milk of Lactating Women From an Urban Area in Central Poland.” Environmental Science and Pollution Research International 28, no. 4: 4549–4557. 10.1007/s11356-020-10767-3. 32946056 PMC7835183

[jsr70286-bib-0038] Groner, J. A. , L. Nicholson , H. Huang , and J. A. Bauer . 2019. “Secondhand Smoke Exposure and Sleep‐Related Breathing Problems in Toddlers.” Academic Pediatrics 19, no. 7: 835–841. 10.1016/j.acap.2019.03.008. 30959225

[jsr70286-bib-0039] Guxens, M. , M. J. Lubczyńska , R. L. Muetzel , et al. 2018. “Air Pollution Exposure During Fetal Life, Brain Morphology, and Cognitive Function in School‐Age Children.” Biological Psychiatry 84, no. 4: 295–303. 10.1016/j.biopsych.2018.01.016. 29530279

[jsr70286-bib-0040] Heudorf, U. , V. Mersch‐Sundermann , and J. Angerer . 2007. “Phthalates: Toxicology and Exposure.” International Journal of Hygiene and Environmental Health 210, no. 5: 623–634. 10.1016/j.ijheh.2007.07.011. 17889607

[jsr70286-bib-0041] Hooper, K. , and T. A. McDonald . 2000. “The PBDEs: An Emerging Environmental Challenge and Another Reason for Breast‐Milk Monitoring Programs.” Environmental Health Perspectives 108: 387. 10.1289/ehp.00108387. 10811563 PMC1638037

[jsr70286-bib-0042] Houde, M. , J. W. Martin , R. J. Letcher , K. R. Solomon , and D. C. G. Muir . 2006. “Biological Monitoring of Polyfluoroalkyl Substances: A Review.” Environmental Science & Technology 40, no. 11: 3463–3473. 10.1021/es052580b. 16786681

[jsr70286-bib-0043] Hoyniak, C. P. , D. J. Whalen , J. L. Luby , et al. 2024. “Sleep and Circadian Rhythms During Pregnancy, Social Disadvantage, and Alterations in Brain Development in Neonates.” Developmental Science 27, no. 3: e13456. 10.1111/desc.13456. 37902111 PMC10997484

[jsr70286-bib-0044] Huang, Y. , F. Fang , Y. Chen , et al. 2023. “Prenatal Exposure to Per‐ and Polyfluoroalkyl Substances and Infant Sleep Disturbance: A Prospective Cohort Study.” Environment International 178: 108070. 10.1016/j.envint.2023.108070. 37399769

[jsr70286-bib-0045] Huang, Y. S. , P. L. Hung , L. J. Wang , et al. 2024. “Distinct Impacts of Prenatal and Postnatal Phthalate Exposure on Behavioral and Emotional Development in Children Aged 1.5 to 3 Years.” Toxics 12, no. 11: 795. 10.3390/toxics12110795. 39590974 PMC11598217

[jsr70286-bib-0046] Iribarne‐Durán, L. M. , F. Artacho‐Cordón , M. Peña‐Caballero , et al. 2019. “Presence of Bisphenol A and Parabens in a Neonatal Intensive Care Unit: An Exploratory Study of Potential Sources of Exposure.” Environmental Health Perspectives 127, no. 11: 117004. 10.1289/EHP5564. 31774309 PMC6927498

[jsr70286-bib-0047] Jacob, J. , and A. Seidel . 2002. “Biomonitoring of Polycyclic Aromatic Hydrocarbons in Human Urine.” Journal of Chromatography B: Analytical Technologies in the Biomedical and Life Sciences 778, no. 1–2: 31–47. 10.1016/s0378-4347(01)00467-4. 12376115

[jsr70286-bib-0048] Jara, S. M. , J. R. Benke , S. Y. Lin , and S. L. Ishman . 2015. “The Association Between Secondhand Smoke and Sleep‐Disordered Breathing in Children: A Systematic Review.” Laryngoscope 125, no. 1: 241–247. 10.1002/lary.24833. 25130300

[jsr70286-bib-0049] Ji, H. , H. Liang , Z. Wang , et al. 2019. “Associations of Prenatal Exposures to Low Levels of Polybrominated Diphenyl Ether (PBDE) With Thyroid Hormones in Cord Plasma and Neurobehavioral Development in Children at 2 and 4 Years.” Environment International 131: 105010. 10.1016/j.envint.2019.105010. 31326823

[jsr70286-bib-0050] Jiang, F. 2020. “Sleep and Early Brain Development.” Annals of Nutrition and Metabolism 75, no. Suppl. 1: 44–54. 10.1159/000508055. 32564032

[jsr70286-bib-0051] Kheirandish‐Gozal, L. , M. Ghalebandi , M. Salehi , M. H. Salarifar , and D. Gozal . 2014. “Neighbourhood Air Quality and Snoring in School‐Aged Children.” European Respiratory Journal 43, no. 3: 824–832. 10.1183/09031936.00113113. 23988771

[jsr70286-bib-0052] Kondo, H. , K. Tanio , Y. Nagaura , et al. 2018. “Sleep Disorders Among Yusho Patients Highly Intoxicated With Dioxin‐Related Compounds: A 140‐Case Series.” Environmental Research 166: 261–268. 10.1016/j.envres.2018.05.033. 29908457

[jsr70286-bib-0053] Korte, J. , K. Wulff , C. Oppe , and R. Siegmund . 2001. “Ultradian and Circadian Activity‐Rest Rhythms of Preterm Neonates Compared to Full‐Term Neonates Using Actigraphic Monitoring.” Chronobiology International 18, no. 4: 697–708. 10.1081/cbi-100106082. 11587091

[jsr70286-bib-0054] Kye, H. , J. Kim , S. Ju , J. Lee , C. Lim , and Y. Yoon . 2023. “Microplastics in Water Systems: A Review of Their Impacts on the Environment and Their Potential Hazards.” Heliyon 9, no. 3: e14359. 10.1016/j.heliyon.2023.e14359. 36950574 PMC10025042

[jsr70286-bib-0055] LaKind, J. S. , M. Goodman , and D. R. Mattison . 2014. “Bisphenol A and Indicators of Obesity, Glucose Metabolism/Type 2 Diabetes and Cardiovascular Disease: A Systematic Review of Epidemiologic Research.” Critical Reviews in Toxicology 44: 121–150. 24392816 10.3109/10408444.2013.860075

[jsr70286-bib-0056] Landrigan, P. , A. Garg , and D. B. J. Droller . 2003. “Assessing the Effects of Endocrine Disruptors in the National Children's Study.” Environmental Health Perspectives 111, no. 13: 1678–1682. 14527850 10.1289/ehp.5799 PMC1241693

[jsr70286-bib-0057] Le, H. H. , E. M. Carlson , J. P. Chua , et al. 2007. “Bisphenol A Is Released From Polycarbonate Drinking Bottles and Mimics the Neurotoxic Actions of Estrogen in Developing Cerebellar Neurons.” Toxicology Letters 176, no. 2: 149–156. 10.1016/j.toxlet.2007.11.001. 18155859 PMC2254523

[jsr70286-bib-0058] Lee, Y. , J. Cho , J. Sohn , and C. Kim . 2023. “Health Effects of Microplastic Exposures: Current Issues and Perspectives in South Korea.” Yonsei Medical Journal 64, no. 5: 301–308. 10.3349/ymj.2023.0048. 37114632 PMC10151227

[jsr70286-bib-0059] Leikauf, G. D. , S. H. Kim , and A. S. Jang . 2020. “Mechanisms of Ultrafine Particle‐Induced Respiratory Health Effects.” Experimental & Molecular Medicine 52, no. 3: 329–337. 10.1038/s12276-020-0394-0. 32203100 PMC7156674

[jsr70286-bib-0060] Leung, A. M. , E. N. Pearce , and L. E. Braverman . 2014. “Environmental Perchlorate Exposure: Potential Adverse Thyroid Effects.” Current Opinion in Endocrinology, Diabetes, and Obesity 21, no. 5: 372–376. 10.1097/MED.0000000000000090. 25106002 PMC4269291

[jsr70286-bib-0061] Lin, L. Z. , J. H. Chen , Y. J. Yu , and G. H. Dong . 2023. “Ambient Air Pollution and Infant Health: A Narrative Review.” eBioMedicine 93: 104609. 10.1016/j.ebiom.2023.104609. 37169689 PMC10363448

[jsr70286-bib-0062] Liu, J. , L. Ghastine , P. Um , E. Rovit , and T. Wu . 2021. “Environmental Exposures and Sleep Outcomes: A Review of Evidence, Potential Mechanisms, and Implications.” Environmental Research 196: 110406. 10.1016/j.envres.2020.110406. 33130170 PMC8081760

[jsr70286-bib-0063] Lobermeier, M. , A. D. Staples , C. Peterson , et al. 2022. “Cumulative Risk, Infant Sleep, and Infant Social‐Emotional Development.” Infant Behavior & Development 67: 101713. 10.1016/j.infbeh.2022.101713. 35339929 PMC9526438

[jsr70286-bib-0064] Lorenzetti, S. , T. Plösch , and I. C. Teller . 2021. “Antioxidative Molecules in Human Milk and Environmental Contaminants.” Antioxidants 10, no. 4: 550. 10.3390/antiox10040550. 33916168 PMC8065843

[jsr70286-bib-0065] Louis, J. , C. Cannard , H. Bastuji , and M. J. Challamel . 1997. “Sleep Ontogenesis Revisited: A Longitudinal 24‐Hour Home Polygraphic Study on 15 Normal Infants During the First Two Years of Life.” Sleep 20, no. 5: 323–333. 10.1093/sleep/20.5.323. 9381053

[jsr70286-bib-0066] Mallah, M. A. , L. Changxing , M. A. Mallah , et al. 2022. “Polycyclic Aromatic Hydrocarbon and Its Effects on Human Health: An Overeview.” Chemosphere 296: 133948. 10.1016/j.chemosphere.2022.133948. 35151703

[jsr70286-bib-0067] Mirmiran, M. , Y. G. H. Maas , and R. L. Ariagno . 2003. “Development of Fetal and Neonatal Sleep and Circadian Rhythms.” Sleep Medicine Reviews 7, no. 4: 321–334. 10.1053/smrv.2002.0243. 14505599

[jsr70286-bib-0068] Mlyczyńska, E. , A. Bongrani , C. Rame , et al. 2023. “Concentration of Polycyclic Aromatic Hydrocarbons (PAHs) in Human Serum and Adipose Tissues and Stimulatory Effect of Naphthalene in Adipogenesis in 3T3‐L1 Cells.” International Journal of Molecular Sciences 24, no. 2: 1455. 10.3390/ijms24021455. 36674971 PMC9861916

[jsr70286-bib-0069] O'Callaghan, F. V. , A. Al Mamun , M. O'Callaghan , et al. 2010. “The Link Between Sleep Problems in Infancy and Early Childhood and Attention Problems at 5 and 14 Years: Evidence From a Birth Cohort Study.” Early Human Development 86, no. 7: 419–424. 10.1016/j.earlhumdev.2010.05.020. 20646881

[jsr70286-bib-0070] Organisation Mondiale de la Sante . 2019. Le Message de l'OMS au Jeune Enfant: Pour Grandir en Bonne Santé, ne Pas Trop Rester Assis et Jouer Davantage.

[jsr70286-bib-0071] Papadopoulos, D. , and F. A. E. Sosso . 2023. “Socioeconomic Status and Sleep Health: A Narrative Synthesis of 3 Decades of Empirical Research.” Journal of Clinical Sleep Medicine 19, no. 3: 605–620. 10.5664/jcsm.10336. 36239056 PMC9978435

[jsr70286-bib-0072] Paquette, A. G. , S. Lapehn , S. Freije , et al. 2023. “Placental Transcriptomic Signatures of Prenatal Exposure to Hydroxy‐Polycyclic Aromatic Hydrocarbons.” Environment International 172: 107763. 10.1016/j.envint.2023.107763. 36689866 PMC10211546

[jsr70286-bib-0073] Patel, M. , R. Kumar , K. Kishor , T. Mlsna , C. U. Pittman Jr. , and D. Mohan . 2019. “Pharmaceuticals of Emerging Concern in Aquatic Systems: Chemistry, Occurrence, Effects, and Removal Methods.” Chemical Reviews 119, no. 6: 3510–3673. 10.1021/acs.chemrev.8b00299. 30830758

[jsr70286-bib-0074] Payne‐Sturges, D. C. , T. K. Taiwo , K. Ellickson , et al. 2023. “Disparities in Toxic Chemical Exposures and Associated Neurodevelopmental Outcomes: A Scoping Review and Systematic Evidence Map of the Epidemiological Literature.” Environmental Health Perspectives 131, no. 9: 96001. 10.1289/EHP11750. 37754677 PMC10525348

[jsr70286-bib-0075] Peirano, P. , I. Fagioli , F. Bes , and P. I. E. R. O. Salzarulo . 1993. “The Role of Slow‐Wave Sleep on the Duration of Quiet Sleep in Infants.” Journal of Sleep Research 2, no. 3: 130–133. 10.1111/j.1365-2869.1993.tb00075.x. 10607083

[jsr70286-bib-0076] Prüst, M. , J. Meijer , and R. H. S. Westerink . 2020. “The Plastic Brain: Neurotoxicity of Micro‐ and Nanoplastics.” Particle and Fibre Toxicology 17, no. 1: 24. 10.1186/s12989-020-00358-y. 32513186 PMC7282048

[jsr70286-bib-0077] Ramírez Benítez, Y. , M. Díaz Bringas , R. M. Jiménez‐Morales , I. B. Ngyah‐Etchutambe , and L. S. Pagani . 2025. “Secondhand Smoke Exposure and Brain Health Indicators in Cuban Preschoolers.” Toxics 13, no. 1: 62. 10.3390/toxics13010062. 39853060 PMC11768478

[jsr70286-bib-0078] Rice, D. , and S. Barone . 2000. “Critical Periods of Vulnerability for the Developing Nervous System: Evidence From Humans and Animal Models.” Environmental Health Perspectives 108, no. Suppl 3: 511–533. 10.1289/ehp.00108s3511. 10852851 PMC1637807

[jsr70286-bib-0079] Roffwarg, H. P. , J. N. Muzio , and W. C. Dement . 1966. “Ontogenetic Development of the Human Sleep‐Dream Cycle.” Science 152, no. 3722: 604–619. 10.1126/science.152.3722.604. 17779492

[jsr70286-bib-0080] Sadeh, A. , G. De Marcas , Y. Guri , A. Berger , L. Tikotzky , and Y. Bar‐Haim . 2015. “Infant Sleep Predicts Attention Regulation and Behavior Problems at 3–4 Years of Age.” Developmental Neuropsychology 40, no. 3: 122–137. 10.1080/87565641.2014.973498. 26151611

[jsr70286-bib-0081] Sánchez, T. , D. Gozal , D. L. Smith , C. Foncea , C. Betancur , and P. E. Brockmann . 2019. “Association Between Air Pollution and Sleep Disordered Breathing in Children.” Pediatric Pulmonology 54, no. 5: 544–550. 10.1002/ppul.24256. 30719878

[jsr70286-bib-0082] Sears, C. G. , and J. M. Braun . 2021. “Urinary Phthalate Metabolite Concentrations and Adolescent Sleep Duration.” Environmental Epidemiology 5, no. 2: e134. 10.1097/EE9.0000000000000134. 33870010 PMC8043726

[jsr70286-bib-0083] Serrano‐Nascimento, C. , and M. T. Nunes . 2022. “Perchlorate, Nitrate, and Thiocyanate: Environmental Relevant NIS‐Inhibitors Pollutants and Their Impact on Thyroid Function and Human Health.” Frontiers in Endocrinology 13: 995503. 10.3389/fendo.2022.995503. 36339434 PMC9633673

[jsr70286-bib-0084] Sharma, J. , M. Joshi , A. Bhatnagar , A. K. Chaurasia , and S. Nigam . 2022. “Pharmaceutical Residues: One of the Significant Problems in Achieving ‘*Clean Water for All*’ and Its Solution.” Environmental Research 215: 114219. 10.1016/j.envres.2022.114219. 36057333

[jsr70286-bib-0085] Shukla, A. , N. Bunkar , R. Kumar , et al. 2019. “Air Pollution Associated Epigenetic Modifications: Transgenerational Inheritance and Underlying Molecular Mechanisms.” Science of the Total Environment 656: 760–777. 10.1016/j.scitotenv.2018.11.381. 30530146

[jsr70286-bib-0086] Siegel, J. M. 2004. “The Neurotransmitters of Sleep.” Journal of Clinical Psychiatry 65, no. Suppl 16: 4. PMC8761080 15575797

[jsr70286-bib-0087] Simeone, R. M. , P. P. Howards , E. Anderson , et al. 2022. “Pre‐ and Postnatal Polychlorinated Biphenyl Exposure and Cognitive and Behavioral Development at Age 45 Months in a Cohort of Slovak Children.” Chemosphere 287, no. 4: 132375. 10.1016/j.chemosphere.2021.132375. 34597632 PMC8629853

[jsr70286-bib-0088] Singh, V. K. , D. K. Patel , S. Ram , N. Mathur , M. K. Siddiqui , and J. R. Behari . 2008. “Blood Levels of Polycyclic Aromatic Hydrocarbons in Children of Lucknow, India.” Archives of Environmental Contamination and Toxicology 54, no. 2: 348–354. 10.1007/s00244-007-9015-3. 17763887

[jsr70286-bib-0089] Stefanak, M. P. , F. Al‐Mudares , D. El‐Metwally , J. W. Jones , M. A. Kane , and C. F. Bearer . 2020. “High Concentrations of Urinary Ethanol Metabolites in Neonatal Intensive Care Unit Infants.” Pediatric Research 88, no. 6: 865–870. 10.1038/s41390-020-1020-5. 32563185

[jsr70286-bib-0090] Stéphan‐Blanchard, E. , K. Chardon , D. D. Djeddi , et al. 2016. “The Dynamics of Cardiac Autonomic Control in Sleeping Preterm Neonates Exposed *in Utero* to Smoking.” Clinical Neurophysiology 127, no. 8: 2871–2877. 10.1016/j.clinph.2016.05.001. 27246968

[jsr70286-bib-0091] Stockholm . 2001. Stockholm Convention on Persistent Organic Pollutants.

[jsr70286-bib-0092] Terry, A. V. 2012. “Functional Consequences of Repeated Organophosphate Exposure: Potential Non‐Cholinergic Mechanisms.” Pharmacology & Therapeutics 134, no. 3: 355–365. 10.1016/j.pharmthera.2012.03.001. 22465060 PMC3366364

[jsr70286-bib-0094] Tham, E. K. , N. Schneider , and B. F. Broekman . 2017. “Infant Sleep and Its Relation With Cognition and Growth: A Narrative Review.” Nature and Science of Sleep 9: 135–149. 10.2147/NSS.S125992. PMC5440010 28553151

[jsr70286-bib-0095] Thompson, L. A. , W. S. Darwish , Y. Ikenaka , S. M. M. Nakayama , H. Mizukawa , and M. Ishizuka . 2017. “Organochlorine Pesticide Contamination of Foods in Africa: Incidence and Public Health Significance.” Journal of Veterinary Medical Science 79, no. 4: 751–764. 10.1292/jvms.16-0214. 28302941 PMC5402199

[jsr70286-bib-0096] Tonacchera, M. , A. Pinchera , A. Dimida , et al. 2004. “Relative Potencies and Additivity of Perchlorate, Thiocyanate, Nitrate, and Iodide on the Inhibition of Radioactive Iodide Uptake by the Human Sodium Iodide Symporter.” Thyroid 14, no. 12: 1012–1019. 10.1089/thy.2004.14.1012. 15650353

[jsr70286-bib-0097] Touchette, E. , G. Fréchette‐Boilard , D. Petit , et al. 2024. “Longitudinal Study of Childhood Sleep Trajectories and Adolescent Mental Health Problems.” SLEEP Advances 5, no. 1: zpae013. 10.1093/sleepadvances/zpae013. 38559775 PMC10981463

[jsr70286-bib-0098] Tsou, P. Y. , S. Gueye‐Ndiaye , K. L. Gorman , et al. 2024. “Asthma and Sleep Disordered Breathing in the Pediatric Adenotonsillectomy Trial for Snoring Study.” Sleep & Breathing 29, no. 1: 46. 10.1007/s11325-024-03210-1. 39633037

[jsr70286-bib-0099] Vizcaino, E. , J. O. Grimalt , A. Fernández‐Somoano , and A. Tardon . 2014. “Transport of Persistent Organic Pollutants Across the Human Placenta.” Environment International 65: 107–115. 10.1016/j.envint.2014.01.004. 24486968

[jsr70286-bib-0100] Vork, K. L. , R. L. Broadwin , and R. J. Blaisdell . 2007. “Developing Asthma in Childhood From Exposure to Secondhand Tobacco Smoke: Insights From a Meta‐Regression.” Environmental Health Perspectives 115, no. 10: 1394–1400. 10.1289/ehp.10155. 17938726 PMC2022647

[jsr70286-bib-0101] Wafula, S. T. , L. N. Namakula , J. B. Isunju , et al. 2025. “Association Between Early Life Second‐Hand Smoke Exposure on Child Sleep and Psychoactive Substance Use on Adult Sleep Patterns in an Urban Informal Settlement in Uganda.” PLoS One 20, no. 1: e0312127. 10.1371/journal.pone.0312127. 39752375 PMC11698410

[jsr70286-bib-0102] Wallace, D. A. , J. P. Gallagher , S. R. Peterson , et al. 2023. “Is Exposure to Chemical Pollutants Associated With Sleep Outcomes? A Systematic Review.” Sleep Medicine Reviews 70: 101805. 10.1016/j.smrv.2023.101805. 37392613 PMC10528206

[jsr70286-bib-0103] Wang, Q. , X. Gu , Y. Liu , et al. 2023. “Insights Into the Circadian Rhythm Alterations of the Novel PFOS Substitutes F‐53B and OBS on Adult Zebrafish.” Journal of Hazardous Materials 448: 130959. 10.1016/j.jhazmat.2023.130959. 36860044

[jsr70286-bib-0104] World Health Organization . 1949. “World Health Organization—Regional Office for the Eastern Mediterranean.” In Second‐Hand Smoke Impacts Health. WHO.

[jsr70286-bib-0105] World Health Organization , ed. 2018. WHO Global Report on Trends in Prevalence of Tobacco Smoking 2000–2025. 2nd ed, 120. World Health Organization.

[jsr70286-bib-0106] Xie, Z. , J. Tan , G. Fang , et al. 2022. “Associations Between Prenatal Exposure to Perfluoroalkyl Substances and Neurobehavioral Development in Early Childhood: A Prospective Cohort Study.” Ecotoxicology and Environmental Safety 241: 113818. 10.1016/j.ecoenv.2022.113818. 35777342

[jsr70286-bib-0107] Yang, D. , H. Shi , L. Li , J. Li , K. Jabeen , and P. Kolandhasamy . 2015. “Microplastic Pollution in Table Salts From China.” Environmental Science & Technology 49, no. 22: 13622–13627. 10.1021/acs.est.5b03163. 26486565

[jsr70286-bib-0108] Yin, X. , Y. Liu , R. Zeb , F. Chen , H. Chen , and K.‐J. Wang . 2020. “The Intergenerational Toxic Effects on Offspring of Medaka Fish *Oryzias melastigma* From Parental Benzo[a]Pyrene Exposure via Interference of the Circadian Rhythm.” Environmental Pollution 267: 115437. 10.1016/j.envpol.2020.115437. 32866872

[jsr70286-bib-0109] Yolton, K. , Y. Xu , J. Khoury , et al. 2010. “Associations Between Secondhand Smoke Exposure and Sleep Patterns in Children.” Pediatrics 125, no. 2: e261–e268. 10.1542/peds.2009-0690. 20083521 PMC4900537

[jsr70286-bib-0110] Yolton, K. , Y. Xu , H. Sucharew , et al. 2013. “Impact of Low‐Level Gestational Exposure to Organophosphate Pesticides on Neurobehavior in Early Infancy: A Prospective Study.” Environmental Health 12, no. 1: 79. 10.1186/1476-069X-12-79. 24034442 PMC3848803

[jsr70286-bib-0111] Zamora, A. N. , K. E. Peterson , M. M. Téllez‐Rojo , et al. 2022. “Urinary Phthalates, Phenols, and Parabens in Relation to Sleep Health Markers Among a Cohort of Mexican Adolescents.” Science of the Total Environment 861: 160651. 10.1016/j.scitotenv.2022.160651. 36473659 PMC9880990

[jsr70286-bib-0112] Zamora, A. N. , D. J. Watkins , K. E. Peterson , et al. 2021. “Prenatal Maternal Pesticide Exposure in Relation to Sleep Health of Offspring During Adolescence.” Environmental Research 204: 111977. 10.1016/j.envres.2021.111977. 34469742 PMC8639673

[jsr70286-bib-0113] Zhang, L. , C. Ou , D. Magana‐Arachchi , et al. 2021. “Indoor Particulate Matter in Urban Households: Sources, Pathways, Characteristics, Health Effects, and Exposure Mitigation.” International Journal of Environmental Research and Public Health 18, no. 21: 11055. 10.3390/ijerph182111055. 34769574 PMC8582694

[jsr70286-bib-0114] Zhang, Q. F. , H. H. Bao , W. K. Wu , et al. 2018. “Association Between Early Pregnancy Bisphenol A Exposure and Sleep Problems Among Preschool Children.” Chinese Journal of Preventive Medicine 52, no. 10: 1018–1022. 10.3760/cma.j.issn.0253-9624.2018.10.010. 30392320

[jsr70286-bib-0115] Zhao, H. , L. Fang , Y. Chen , J. Ni , X. Chen , and F. Pan . 2024. “Independent and Combined Associations of Polycyclic Aromatic Hydrocarbons Exposure and Sleep Disorders Among Adults in the U.S. Adult Population.” Journal of Affective Disorders 350: 319–327. 10.1016/j.jad.2024.01.111. 38220115

